# A Case of Euglycemic Diabetic Ketoacidosis in a Patient With Type 2 Diabetes Mellitus and COVID-19

**DOI:** 10.7759/cureus.12029

**Published:** 2020-12-11

**Authors:** Nathan Morrison, Katherine Barnett, Julianna Tantum, Hannah K Morrison, Michael Whalen

**Affiliations:** 1 Internal Medicine, Philadelphia College of Osteopathic Medicine, Philadelphia, USA; 2 Internal Medicine, Lankenau Medical Center, Wynnewood, USA; 3 Emergency Medicine, Lankenau Medical Center, Wynnewood, USA

**Keywords:** medical intensive care unit, covid 19, sars-cov-2, euglycemic dka, diabetic ketoacidosis (dka), emergency department, diabetes mellitus type 2

## Abstract

Diabetic ketoacidosis (DKA) can cause significant morbidity and mortality in patients with type 1 or type 2 diabetes mellitus. DKA causes an approximate annual hospitalization rate of 6.3% and in-hospital case-fatality rate of 0.4%. A subset of DKA cases termed euglycemic diabetic ketoacidosis (eu-DKA) is characterized by euglycemia (<200 mg/dL), high anion gap metabolic acidosis, and an increased plasma ketone concentration. This clinical syndrome comprises approximately 2.6% to 3.2% of total DKA admissions, making it a rare condition. In this case report, a male patient was diagnosed with coronavirus disease 2019 (COVID-19) three days prior to arriving at the emergency department. Upon evaluation, he displayed severe acidemia and was diagnosed with eu-DKA. He was started on intravenous regular insulin and D5 one-half normal saline, which markedly improved his metabolic status. Notably, his admission was uncomplicated by respiratory symptoms of COVID-19. It is proposed that his eu-DKA was catalyzed by his recent COVID-19 infection. Recent studies that have shown COVID-19 may increase lipolysis and induce ketogenesis in susceptible patients.

## Introduction

The Centers for Disease Control and Prevention (CDC) has indicated that adults with type 2 diabetes mellitus (T2DM) are at an increased risk of severe morbidity and mortality with coronavirus disease 2019 (COVID-19) infections [1]. A life-threatening complication of diabetes, known as diabetic ketoacidosis (DKA), causes an approximate annual hospitalization rate of 6.3% and in-hospital case-fatality rate of 0.4% [2]. DKA is characterized by a triad including uncontrolled hyperglycemia (>250 mg/dL), high anion gap metabolic acidosis, and an increased plasma ketone concentration. However, DKA can rarely occur with a normal blood glucose level (<200 mg/dL), which is termed euglycemic DKA (eu-DKA). An estimated 2.6% to 3.2% of DKA admissions are eu-DKA cases [3-5]. This case report reviews the hospital course of a patient who presented to the emergency department (ED) with eu-DKA and severe acidemia in the setting of a known COVID-19 infection for three days. The patient was also treated for his T2DM with an sodium-glucose cotransporter 2 (SGLT2) inhibitor. The rare incidence of eu-DKA causes the possibility of misdiagnosis in patients, and the need for increased awareness in the midst of the COVID-19 pandemic. A recent study by Li et al. recognized COVID-19 as a possible cause of ketosis and ketoacidosis in susceptible patients [6].

## Case presentation

**Figure 1:**
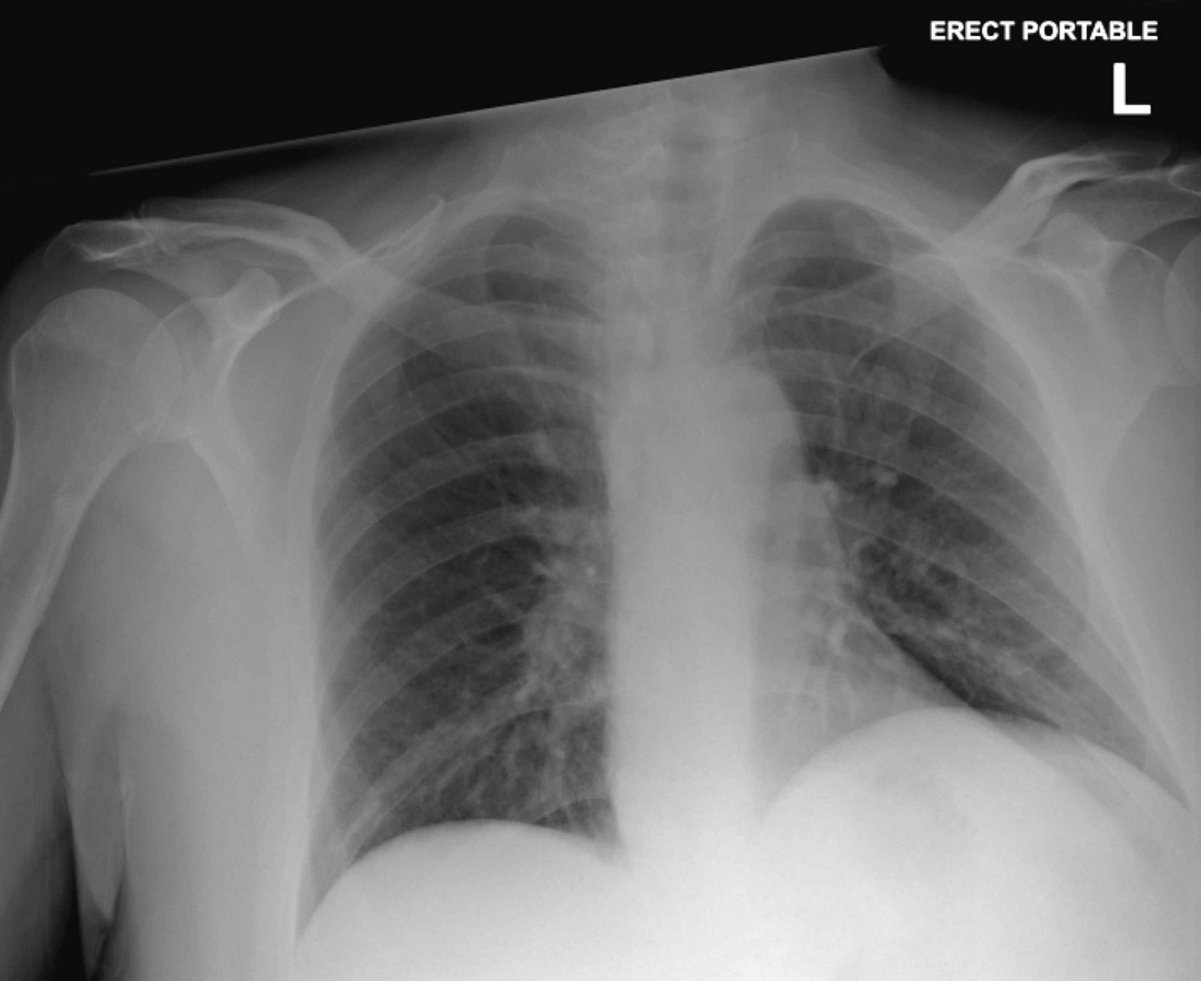
A single anteroposterior view of the chest via chest x-ray No acute disease is displayed. There are signs of degenerative changes in the shoulders.

A male in his 40s with a past medical history of T2DM and hyperlipidemia was diagnosed with COVID-19 three days prior to presentation to the ED. The patient came for the evaluation of worsening symptoms of fatigue, non-productive cough, and poor appetite for six days. He also admitted to several episodes of non-bloody, non-bilious vomiting, and poor oral intake. When questioned, he reported compliance with his home medications including 25 mg of empagliflozin daily, 3 mg of semaglutide daily, 500 mg of metformin twice per day, 40 mg of atorvastatin daily, and 200 mg of modafinil daily. On physical examination, significant findings showed that he was diaphoretic and actively vomiting. His vitals signs were significant for a heart rate of 113 beats per minute, temperature of 37.6 °C (99.7 °F), blood pressure of 122/95 mm Hg, 20 breaths per minute, and 97% oxygen saturation on room air. Comprehensive metabolic panel (CMP) results were notable for a serum sodium level of 133 mEq/L (corrected sodium of 134 mEq/L), carbon dioxide 11 mEq/L, creatinine of 1.5 mg/dL, glucose 177 mg/dL, and calculated anion gap of 25 mEq/L. Complete blood count (CBC) results were significant for a white blood cell count of 11.76 × 10^3^/μL and red blood cell count of 20.4 × 10^6^/μL. Beta-hydroxybutyrate resulted in 8.62 mmol/dL. Venous blood gas (VBG) determined a pH of 7.06, partial pressure of carbon dioxide of 37 mm Hg, partial pressure of oxygen of 31 mm Hg, bicarbonate level of 10.0 mEq/L, and lactate 2.3 mmol/L. Urinalysis was positive for glucose (≥1000 mg/dL) and ketones (>80 mg/dL). Other non-specific laboratory results for active COVID-19 infection revealed a lactate dehydrogenase level of 259 U/L, ferritin 591 ng/mL, creatine kinase 59 U/L, and C-reactive protein level of 94.2 mg/L. His chest x-ray was negative for acute disease (Figure 1). Upon initial evaluation in the ED, the patient was administered 2 L of normal saline, 4 mg of intravenous ondansetron, and 20 mg of intravenous famotidine. After laboratory evaluation and diagnosis of eu-DKA, he was started on continuous intravenous (IV) regular insulin at a rate of 1.5 units/hour and a continuous IV infusion of D5 one-half normal saline at a rate of 100 mL/hour. The patient was admitted to the medical ICU for further management of eu-DKA in the setting of active COVID-19 infection. The patient’s hemoglobin A1c was determined to be 10.6% at admission. While in the ICU, the patient was continued on the same regimen of IV insulin and D5 one-half normal saline that was started earlier in the ED. His bicarbonate improved to 22 mEq/L and his anion gap normalized to 10 mEq/L. He was transferred to the general medicine floor after three days, and his regimen was transitioned to 25 units of subcutaneous insulin glargine every night and 10 units of subcutaneous insulin lispro before meals, as recommended by endocrinology. Four days later, he was discharged home in a stable condition and instructed to follow up with endocrinology for his eu-DKA episode and T2DM management. Throughout his entire hospital admission, his COVID-19 respiratory symptoms remained fairly mild.

## Discussion

Adults with T2DM are at an increased risk of contracting severe illness and mortality with COVID-19 infections [1]. In this case, the patient presented to the ED with vomiting and a worsening non-productive cough. He was diagnosed with eu-DKA in the setting of a positive COVID-19 infection for three days.

There have been three other reported cases of patients with a history of diabetes that presented with eu-DKA in the setting of active COVID-19 infection. In one case, the patient had a history of type 1 diabetes mellitus (T1DM), and his admission was complicated by COVID-19 pneumonia and mechanical ventilation. This case did not report a measured serum pH [7]. In the second case, the patient had a history of T2DM, showed signs of COVID-19 pneumonia on imaging, had decreased oxygen saturation on room air, required high flow nasal cannula for eight days, and displayed moderate acidemia in the ICU (pH = 7.28) [8]. The third case had a patient with a history of T2DM who also showed signs of COVID-19 pneumonia on imaging, resulting hypoxemic respiratory failure with intubation, and moderate acidemia (pH = 7.24) [9].

To our knowledge, this is the only case of a patient with a medical history of T2DM treated with an SGLT2 inhibitor, a COVID-19 diagnosis, severe acidemia (pH ≤ 7.1), negative imaging, and no signs of respiratory compromise [7-11]. In this patient, it is proposed that the severe acidemia (pH = 7.06) was exacerbated by his active COVID-19 infection, causing eu-DKA. A study by Li et al. suggested that COVID-19 may increase lipolysis and induce ketosis [6]. This finding suggested that patients with diabetes may be susceptible to DKA with active COVID-19 infections. Noticeably, interleukin-6 (IL-6) is found to be markedly elevated by COVID-19 and is related to its prognosis. IL-6 is a proposed dynamic cause of ketone production through the induction of cellular insulin resistance in hepatocytes [4,12-14]. In this case, it is possible that the severe acidemia and eu-DKA were exacerbated by his COVID-19 symptoms that began six days earlier. Although SGLT-2 inhibitors have been related to eu-DKA cases, it was determined a less likely precipitating etiology in this patient due to the recent infectious diagnosis. According to the Empagliflozin Cardiovascular Outcome Event Trial in Type 2 diabetes Mellitus Patients (EMPA-REG Outcome Trial), his use of empagliflozin is known to be an extremely rare cause of eu-DKA. It was found that cases of DKA related to the medication are around 0.1% [3-5]. It is also possible his empagliflozin therapy could have played a synergistic role with COVID-19 in his eu-DKA presentation through medication-induced glycosuria, decreasing his plasma glucose and increasing ketogenesis [4].

## Conclusions

The rare presentation and prevalence of eu-DKA causes the possibility of misdiagnosis in patients, and the need for increased awareness in the center of the COVID-19 pandemic. Recent studies have recognized COVID-19 as a possible cause of ketosis and ketoacidosis in susceptible patients. Therefore, surveillance of diabetic patients for plausible eu-DKA should be warranted with a COVID-19 suspicion or diagnosis. The diagnosis eu-DKA may go unrecognized in susceptible patients if neglected in an acute care setting.
